# 4-Hydroxychalcone attenuates AngII-induced cardiac remodeling and dysfunction via regulating PI3K/AKT pathway

**DOI:** 10.1038/s41440-024-02068-w

**Published:** 2024-12-24

**Authors:** Xiao Han, Qian-Qiu Zhu, Zhi Li, Jia-Kang He, Yan Sun, Qing-Hua Zhong, Sheng-Xing Tang, Yun-Long Zhang

**Affiliations:** 1 Institute of Cardio-Cerebrovascular Medicine, Central Hospital of Dalian University of Technology, No.826, South West Road, Shahekou District, Dalian, 116089 China; 2 Department of Cardiology, Yijishan Hospital of Wannan Medical College, No. 2, Zheshan West Road, Wuhu, 241000 China; 3 Department of Cardiology, First Affiliated Hospital of Dalian Medical University, No.193, Lianhe Road, Xigang District, Dalian, 116011 China; 4 Department of Cardiology, Longgang Central Hospital of Shenzhen, No.6802, Longgang Road, Longgang District, Shenzhen, 518000 China

**Keywords:** Angiotensin II, Cardiac remodeling, 4-Hydroxychalcone, Network pharmacology, PI3K-AKT

## Abstract

Cardiac remodeling encompasses structural alterations such as hypertrophy, fibrosis, and dilatation, alongside numerous cellular and molecular functional aberrations, constituting a pivotal process in the advancement of heart failure (HF). 4-Hydroxychalcone (4-HCH) is a class of naturally occurring compounds with variable phenolic structures, and has demonstrated the preventive efficacy in hyperaldosteronism, inflammation and renal injury. However, the role of 4-HCH in the regulation of cardiac remodeling remains uncertain. A cardiac remodeling model was established in male C57BL/6 J mice via subcutaneous Ang II (1000 or 300 ng/kg/min) for 2 weeks. Mice were treated with 4-HCH (20 or 40 mg/kg/day) or vehicle control. Systolic blood pressure (SBP) was measured using a tail-cuff method, and echocardiography assessed cardiac function. Histopathological staining evaluated cardiomyocyte hypertrophy, fibrosis, inflammation, and superoxide production. Network pharmacology analysis identified potential core targets and pathways mediating the effects of 4-HCH. Expression of inflammatory cytokines and proteins related to hypertrophy, fibrosis, inflammation, and oxidative stress was assessed by quantitative real-time PCR (qPCR) and Western blotting. Our results indicated that 4-HCH significantly ameliorated Ang II-induced hypertension, cardiomyocyte hypertrophy, fibroblast activation, fibrosis, inflammation, superoxide production, and cardiac function. Network pharmacology analysis identified the PI3K-AKT pathway as a crucial mechanism underlying the effects of 4-HCH, with experimental verification demonstrating that it inhibits cardiac remodeling by downregulating this pathway and its downstream effectors, including mTOR/ERK, TGF-β/Smad2/3, NF-κB, and NOX1 independent of its blood pressure-lowering effects. These results reveal for the first time that 4-HCH alleviates cardiac remodeling, emphasizing its potential as a therapeutic agent for HF.

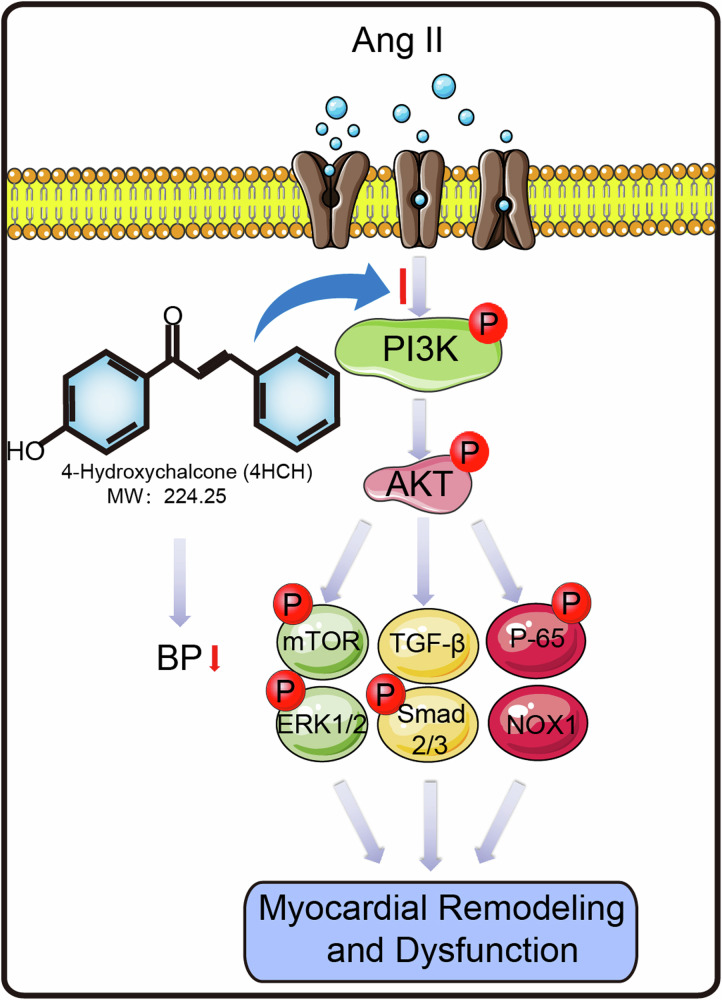

## Introduction

Cardiac remodeling refers to the structural and functional alterations that occur in the left ventricle in response to pathophysiological stimuli, such as hypertension, ischemia/reperfusion or pressure overload. This process serves as a precursor to heart failure (HF) [1, 2]. Cardiac remodeling involves cardiomyocyte hypertrophy, apoptosis, necrosis, as well as myofibroblast activation, transdifferentiation and elevation of fibrillar collagen [2]. Both experimental and clinical evidence indicate that cardiac remodeling may be reversible [3, 4]. Therefore, elucidating the mechanisms underlying cardiac remodeling is essential for the development of effective antifibrotic strategies.

A growing body of evidence suggests that multiple factors, including Angiotensin II (Ang II), pressure overload, high-salt intake, and isoprenaline (ISO), modulate the pathogenesis of cardiac remodeling by activating various molecular pathways such as AKT/mTOR/ERK, JAK/STAT, NF-κB, and TGF-β/Smad, ultimately leading to cardiac remodeling and dysfunction [5, 6] Despite extensive exploration of the specific mechanisms underlying pathological myocardial remodeling, effective treatments remain elusive. Recent studies indicate that natural flavonoid compounds may provide protective effects against cardiovascular diseases [7]; however, the role of 4-Hydroxychalcone (4-HCH) in Angiotensin II-induced cardiac remodeling remains unknown.

4-HCH is an α,β-unsaturated ketone characterized by a chalcone core structure with a hydroxyl substituent at the 4-position of the A ring, which is regarded as a precursor to flavonoids and isoflavones in plants [8]. 4-HCH demonstrates a diverse array of biological properties, including antioxidant, anti-inflammatory, and anti-angiogenic activities [9, 10]. A previous study demonstrated that 4-HCH mitigates hyperaldosteronism, inflammation, and renal injury by inhibiting TNF-α-induced activation of the NF-κB pathway in mice [11]. Therefore, we speculate that 4-HCH could produce beneficial effects on hypertensive myocardial remodeling by modulating key signaling pathways.

In this study, we present the first evidence that the administration of 4-HCH effectively prevents Angiotensin II-induced hypertension, cardiac remodeling and dysfunction in mice. Importantly, these protective effects are independent of mechanisms associated with BP reduction. Based on web-based pharmaceutical analysis and experimental validation, this beneficial effect is associated with the inhibition of PI3K/AKT/mTOR/ERK, TGF-β/Smad2/3, NF-κB, and NADPH oxidase signaling pathways. Collectively, these findings suggest that 4-HCH may serve as a promising agent for the treatment of hypertensive cardiac remodeling.

## Material and methods

### Animals, experimental groups, and interventions

Male wild-type (WT) C57BL/6 mice were obtained from SPF Biotechnology Co., Ltd. (Beijing, China). Cardiac remodeling model was established by subcutaneous infusion of Ang II at a dose of 1000 or 300 ng/kg/min as described previously [12]. 4-HCH (Selleck, Houston, TX, USA) was dissolved in DMSO and subsequently administered via intraperitoneal injection at doses of 20 or 40 mg/kg body weight (BW) daily. All experimental mice (*n* = 50) were randomly assigned to five groups: Saline + Vehicle, Saline + 4-HCH (40 mg/kg), Ang II + Vehicle, Ang II + 4-HCH (20 mg/kg), and Ang II + 4-HCH (40 mg/kg). After two weeks of Ang II infusion, the mice were euthanized, and their hearts were excised for further histological and molecular analysis. The ratios of heart weight to body weight (HW/BW) and heart weight to tibial length (HW/TL) were also calculated. All animal experimental protocols were approved by the Animal Care and Use Committee of Wannan Medical College and Dalian Medical University, and conformed to the Guide for the Care and Use of Laboratory Animals published by the US National Institutes of Health.

### Blood pressure measurement and echocardiography evaluation

Blood pressure was monitored using a noninvasive tail-cuff system (BP-2010A; Softron, Tokyo, Japan) every other day, and the average of 10,11 measurements was utilized for data analysis. At two weeks post Angiotensin II infusion, mice were anesthetized with isoflurane (1.5%) and subsequently underwent M-mode echocardiography utilizing a 30-MHz probe (Vevo 1100, VisualSonics, Toronto, Canada). Left ventricular ejection fraction (EF%) and left ventricular fractional shortening (FS%), which characterize systolic function, were recorded [12].Diastolic function can be estimated from the E/A ratio using pulsed-wave Doppler echocardiography, which includes peak early diastolic filling (E wave) and late diastolic filling (A wave) velocities [13].

### Histopathological analysis

The isolated heart samples were fixed in 4% paraformaldehyde for more than 24 h, and then embedded in paraffin. The sections of the cardiac tissues (5 μm thickness) were stained with hematoxylin and eosin, Masson’s trichrome, and wheat germ agglutinin (WGA, Sigma-Aldrich, St. Louis., MO, USA) [12]. Immunohistochemistry was conducted using an α-smooth muscle actin (α-SMA) antibody (Abcam, Cambridge, UK; 1:200 dilution) and Mac-2-antibodies (Santa Cruz, Dallas, Texas, USA; 1:200 dilution) on the heart sections, followed by incubation at 4°C overnight and subsequent binding of the secondary antibody at room temperature for 30 min [14]. To detect reactive oxygen species (ROS), heart cryosections (5 μm thickness) were stained with dihydroethdium (DHE, Sigma-Aldrich, St. Louis., MO, USA) at a concentration of 1 μmol/L in PBS at 37°C for 30 min [14]. All digital images were captured at ×100 or ×200 magnification from over 20 random fields using a Labophot 2 microscope (OLYMPUS, BX53, Japan). The myocyte cross-sectional areas, fibrotic areas, α-SMA-positive areas, and DHE intensity were quantitatively analyzed using ImageJ Software (NIH, Bethesda, MD) as previously described [12].

### Quantitative real-time PCR analysis (qPCR)

Total RNA was isolated and purified from heart tissues using TRIzol (Invitrogen, Carlsbad, CA, USA).cDNA was synthesized using the PrimeScript RT Kit (Yeasen Biotech Co., Shanghai, China), and qPCR was performed using SYBR Green Master Mix (Takara, Kusatsu, Japan) on the Applied Biosystems 7500 Fast System (ABI, Carlsbad, CA, USA). The mRNA levels were quantified relative to GAPDH expression using the 2^ΔΔCt^ method [12]. The primers for IL-6: Forward primer (5’-3’): GCTACCAAACTGGATATAATCAGGA. Reverse primer (5’-3’): CCAGGTAGCTATGGTACTCCAGAA-5. ICAM-1: Forward primer (5’-3’): GTCCGCTGTGCTTTGAGAACT. Reverse primer (5’-3’): CGGAAACGAATACACGGTGAT. GAPDH: Forward primer (5’-3’): GGTTGTCTCCTGCGACTTCA. Reverse primer (5’-3’): GGTGGTCCAGGGTTTCTTACTC.

### Western blot analysis

Total proteins were extracted from heart tissue using RIPA buffer (Solarbio Science Technology Co., Beijing, China). Equal amounts of protein (50 µg) were separated by electrophoresis on 10-15% sodium dodecyl sulfate-polyacrylamide (SDS-PAGE) gels and transferred to polyvinylidene fluoride (PVDF) membranes (Millipore, Billerica, MA, USA). The membranes were incubated with primary antibodies at 4°C overnight and subsequently with secondary antibodies (1:5000) at room temperature for 1 h. GAPDH served as an internal control. The primary antibodies: Phospho-AKT1 (9271S), AKT1 (9272S), Phospho-mTOR (5536S), mTOR (2972S), Phospho-ERK (4370S), ERK (4695S), p-Smad2/3 (8828S) Smad2/3 (8685S), p-P65 (3039), P65 (4764), GAPDH (5174S) were purchased from Cell Signaling Technology (Boston, MA, USA). Phospho-PI3K (bs-3332R) was purchased from Bioss (Beijing, China). PI3K (AF4671) was purchased from Affinity Biosciences (OH, USA). NOX1 (17772-1-AP) was purchased from Proteintech (Rosemont, IL). TGF-β1 (ARG10002) was purchased from Proteintech (Taiwan, China). Anti-mouse and anti-rabbit IgG secondary antibodies were obtained from Cell Signaling Technologies (Boston, MA, USA). Images were captured using Chemiluminescence Imager (Shenhua Science Technology Co.,Ltd., Hangzhou, China). Quantification of Western blots was performed using ImageJ (NIH, Bethesda, Maryland, USA), as previously described [12].

### Aldosterone assay in plasma

Aldosterone (ALD) concentrations were quantified using an Enzyme-Linked Immunosorbent Assay (ELISA) kit (E-EL-0070, Elabscience Biotechnology) in accordance with the manufacturer’s instructions.

### Pharmacological network analysis

Network pharmacology analysis was conducted to investigate the potential targets of 4-HCH using Swiss Target Prediction (http://www.swisstargetprediction.ch/), as previously described [15]. In this study, cardiac remodeling-related targets were identified from GeneCards (https://www.genecards.org/), OMIM (https://www.ncbi.nlm.nih.gov/omim), and DisGeNET (https://www.disgenet.org/) databases. The intersection of 4-HCH-related targets and cardiac remodeling-related targets was determined using a Venn diagram, resulting in the identification of common targets [16]. The common targets were imported into the STRING database (https://cn.string-db.org) for protein–protein interaction (PPI) analysis. Topological analysis was conducted to assess the significance of nodes, incorporating three parameters: degree, betweenness, and closeness. Furthermore, common targets were utilized for Gene Ontology (GO) and Kyoto Encyclopedia of Genes and Genomes (KEGG) pathway enrichment analyses using the DAVID (https://david.ncifcrf.gov) database [15].

### Statistical analysis

The results are presented as mean ± SEM. Statistical analysis was conducted using the Statistical Package for the Social Sciences (SPSS), Version 24.0 (IBM Corp., Armonk, NY).

The Shapiro-Wilk test was conducted to assess the normality of the data. For comparisons between two groups with normally distributed data, the Student’s *t*-test was employed. In instances where the data did not satisfy the assumption of normality, the Mann-Whitney U test was utilized. Additionally, repeated measures ANOVA was performed to analyze blood pressure outcomes. A *P*-value of less than 0.05 was considered statistically significant.

## Results

### 4-HCH attenuates Ang II-caused hypertension and improves cardiac dysfunction

**Fig. 1:**
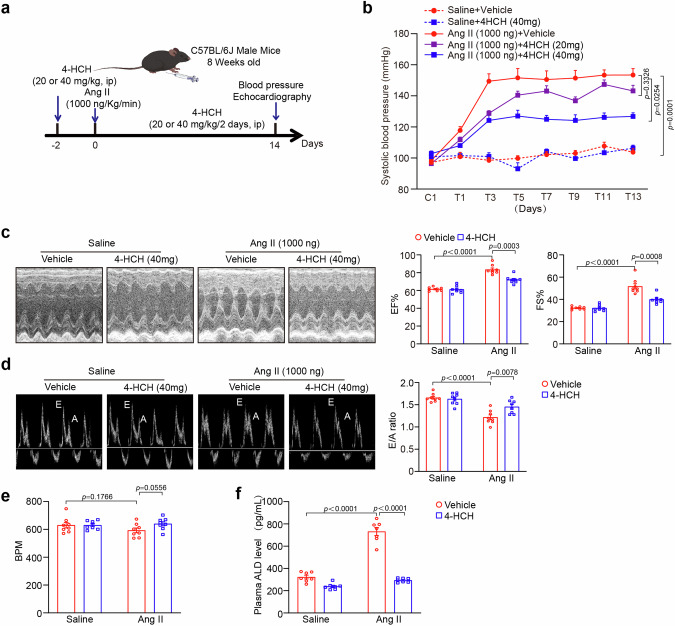
4-HCH ameliorates Ang II-induced hypertension and improves cardiac dysfunction. **a** Wild-type (WT) mice, aged 8 weeks, were subjected to intraperitoneal injection of Ang II (1000 ng/kg/min) for a duration of 2 weeks and received daily administration of 4-HCH at doses of 20 or 40 mg/kg body weight. **b** Systolic blood pressure (SBP) was measured every 2 days using the tail-cuff method (*n* = 6-7). **c** Representative M-mode echocardiography images of the left ventricular chamber (left) and measurements of left ventricular ejection fraction (EF%) and fractional shortening (FS%) (right, *n* = 8). **d** Pulse-wave Doppler images of mitral inflow from the apical 4-chamber view (left) and measurement of the E/A ratio (right, *n* = 8). **e** Measurements of heart rate (*n* = 8). **f** Measurements of plasma aldosterone (ALD) levels (*n* = 7). Data are presented as mean ± SEM, with n representing the number of animals. *P* < *0.05* was considered statistically significant

To evaluate the effect of 4-HCH on cardiac remodeling and function, wild-type (WT) mice were injected intraperitoneally with either vehicle or 4-HCH at doses of 20 or 40 mg/kg daily and subjected to saline or Ang II (1000 ng/kg/min) infusion for a duration of 2 weeks (Fig. 1a). Consistent with our previous findings [12, 14], Angiotensin II infusion significantly elevated the systolic blood pressure (SBP) of the mice compared to saline-treated controls. This increase of SBP was inhibited by 4-HCH at a high dose of 40 mg/kg in Ang II-treated mice (Fig. 1b). Notably, treatment with 4-HCH at a low dose of 20 mg/kg did not yield a significant effect on the angiotensin II-induced elevation of SBP (Fig. 1b). Ang II significantly influences the severity of hypertension by stimulating aldosterone (ALD) secretion [17]. Building on previous studies that demonstrated 4-HCH’s ability to suppress hyperaldosteronism in mice [11], we quantified plasma ALD levels using an ELISA kit. Our results show that 4-HCH effectively mitigates the Ang II-induced elevation of ALD (Fig. 1f), indicating that the antihypertensive effects of 4-HCH are mediated by ALD suppression. Moreover, echocardiography data indicated that Ang II-induced cardiac dysfunction, characterized by an increase in ejection fraction (EF%) and fractional shortening (FS%), as well as a decrease in the transmitral early (E) to atrial (A) wave ratio, was significantly alleviated by 4-HCH at a high dose of 40 mg/kg (Fig. 1c, d). No significant differences in heart rate were observed between the 4-HCH and vehicle-treated groups of mice following Ang II infusion (Fig. 1e). Thus, these results suggest that 4-HCH attenuates angiotensin II-induced hypertension and ameliorates cardiac dysfunction.

### 4-HCH blunts Ang II-induced myocardial hypertrophy and fibrosis

**Fig. 2:**
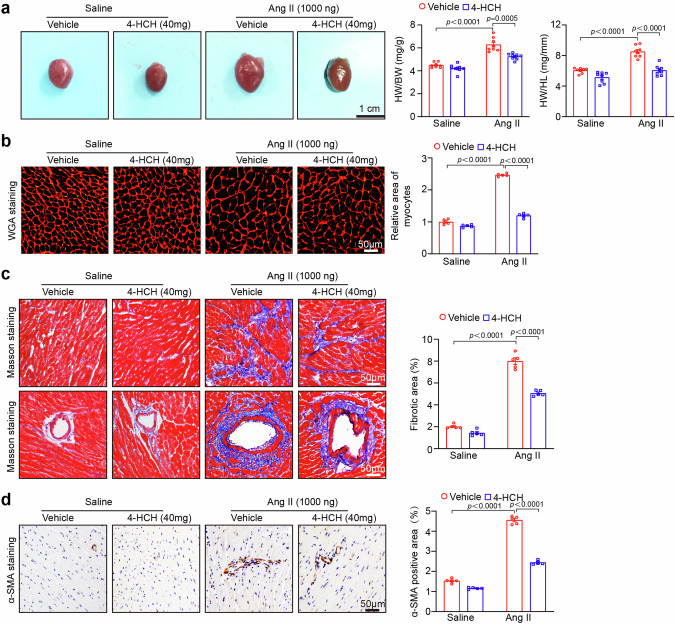
4-HCH reduces Ang II-induced myocardial hypertrophy and fibrosis. WT mice were subjected to intraperitoneal injection of Ang II (1000 ng/kg/min) for a duration of 2 weeks and received daily administration of 4-HCH at doses of 40 mg/kg body weight. **a** Representative images of heart size (left). Scale bar: 5 mm. Ratios of heart weight to body weight (HW/BW) and heart weight to tibia length (HW/TL) for each group (right, *n* = 8). **b** Representative images of TRITC-labeled wheat germ agglutinin (WGA) staining to assess cardiac hypertrophy (left) and quantification of the cross-sectional area of myocytes (100 cells counted per heart, right, *n* = 5). Scale bar: 50 μm. **c** Masson’s trichrome staining of heart sections (left) and quantification of myocardial collagen deposition (right, *n* = 5). **d** Immunohistochemical staining of cardiac myofibroblasts using an anti-α-SMA antibody (left) and quantification of α-SMA-positive cells (right, *n* = 5). Scale bar: 50 μm. Data are presented as mean ± SEM, with n representing the number of animals. *P* < *0.05* was considered statistically significant

Given that myocardial hypertrophy and fibrosis are hallmarks of cardiac remodeling and dysfunction, we subsequently examined the effects of 4-HCH on heart size, cardiomyocyte size, and collagen deposition. After 2 weeks of treatment with angiotensin II (Ang II), wild-type (WT) mice exhibited significant chamber and myocyte hypertrophy, as evidenced by increased heart size and elevated heart weight/body weight (HW/BW), heart weight/tibia length (HW/TL) ratios and left ventricle (LV) cardiomyocyte size compared to saline-treated controls. Notably, these changes were attenuated in Ang II-treated mice that received 4-HCH at a dosage of 40 mg/kg body weight (Fig. 2a, b). Furthermore, Masson’s trichrome staining and immunohistochemistry demonstrated that angiotensin II-induced increases in myocardial peripheral and interstitial fibrosis, as well as the number of α-SMA-positive myofibroblasts, were significantly reduced by 4-HCH at a high dose of 40 mg/kg body weight in Ang II-treated mice (Fig. 2c, d). No significant differences were observed in these parameters between groups treated with or without 4-HCH (Fig. 2a–d). These results suggest that 4-HCH effectively reverses myocardial hypertrophy and fibrosis induced by Ang II infusion.

### 4-HCH suppresses Ang II-induced cardiac inflammation and oxidative stress

**Fig. 3:**
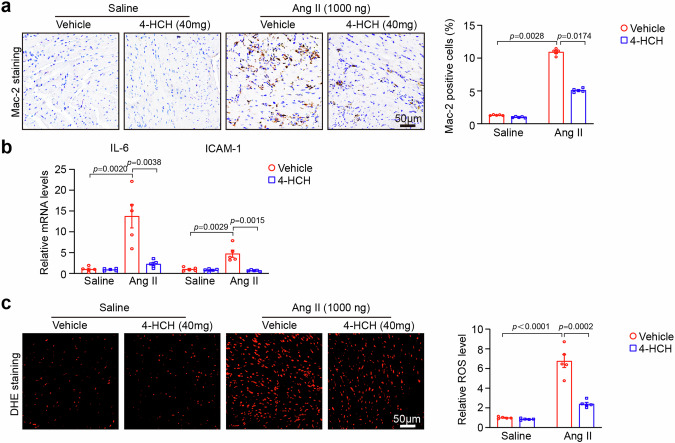
Administration of 4-HCH attenuates Ang II-induced inflammation and oxidative stress. WT mice were subjected to intraperitoneal injection of Ang II (1000 ng/kg/min) for a duration of 2 weeks and received daily administration of 4-HCH at doses of 40 mg/kg body weight. **a** Representative images of Mac-2 staining (left), quantification of Mac-2 positive cells (right, *n* = 5). Scale bar: 50 μm. **b** Quantitative PCR (qPCR) analysis of IL-6 and ICAM-1 gene expression levels (*n* = 5). **c** Dihydroethidium (DHE) staining of heart sections (left) and quantification of DHE fluorescence intensity (right, *n* = 5). Scale bar: 50 μm. Data are presented as mean ± SEM, with n representing the number of animals. *P* < *0.05* was considered statistically significant

We next investigated the effects of 4-HCH on cardiac inflammatory responses and oxidative stress. Immunohistochemical staining for Mac-2 revealed that angiotensin II infusion resulted in a significant increase in the infiltration of Mac-2-positive macrophages, which was markedly reduced in 4-HCH-treated mice (Fig. 3a). Additionally, treatment with 4-HCH significantly decreased the mRNA levels of inflammatory cytokines (IL-6 and ICAM-1) in Ang II-treated mice (Fig. 3b). Dihydroethidium (DHE) staining demonstrated that 4-HCH treatment also significantly lowered reactive oxygen species (ROS) production (Fig. 3c). 4-HCH treatment did not exert a significant effect on these parameters when compared to vehicle treatment in saline-treated controls (Fig. 3a–c).

### 3.4 The protective effects of 4-HCH against Ang II-induced cardiac remodeling are not reliant on mechanisms related to blood pressure (BP) reduction

**Fig. 4:**
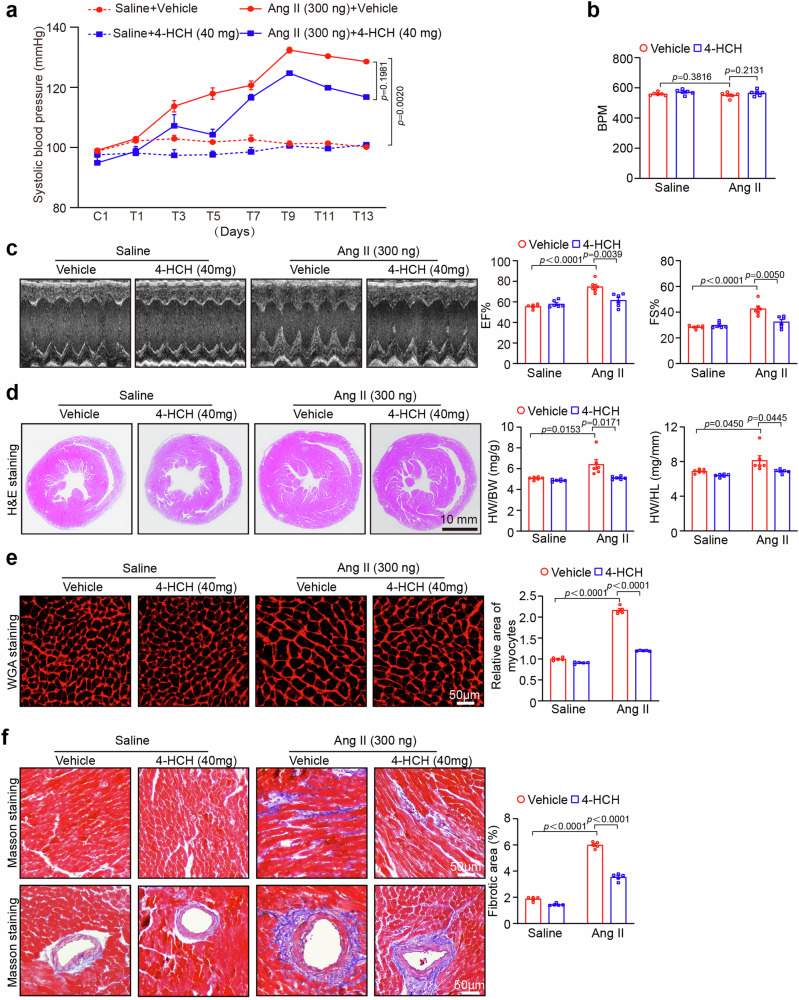
Administration of 4-HCH attenuates cardiac remodeling and dysfunction induced by subpressor infusion of Ang II. **a** WT mice were subjected to intraperitoneal injection of Ang II (300 ng/kg/min) for a duration of 2 weeks and received daily administration of 4-HCH at doses of 40 mg/kg body weight. Systolic blood pressure (SBP) was measured every 2 days using the tail-cuff method (*n* = 6). **b** Measurements of heart rate (*n* = 6). **c** Representative M-mode echocardiography images of the left ventricular chamber (left) and measurements of left ventricular ejection fraction (EF%) and fractional shortening (FS%) (right, *n* = 6). **d** H&E staining of cardiac sections (left). Scale bar: 10 mm. The heart weight to body weight (HW/BW) and heart weight to tibia length (HW/TL) ratios (right, *n* = 6). **e** Representative images of TRITC-labeled wheat germ agglutinin (WGA) staining to assess cardiac hypertrophy (left) and quantification of the cross-sectional area of myocytes (100 cells counted per heart, right, *n* = 5). Scale bar: 50 μm. **f** Masson’s trichrome staining of heart sections (left) and quantification of myocardial collagen deposition (right, *n* = 5). Data are presented as mean ± SEM, with n representing the number of animals. *P* < *0.05* was considered statistically significant

To rule out the possibility that the protective effects of 4-HCH on cardiac remodeling and dysfunction are primarily due to its BP-lowering action, WT mice were intraperitoneally injected with either a vehicle or 4-HCH at a dose of 40 mg/kg daily, followed by infusion with saline or subpressor infusion of Ang II (300 ng/kg/min) for a period of 2 weeks as previously described [18]. We observed that the elevation in SBP was also reduced in 4-HCH (40 mg/kg)-treated mice compared to vehicle controls following Ang II infusion; however, this difference did not reach statistical significance (Fig. 4a).The heart rate was also not significantly different among the groups (Fig. 4b). Conversely, the treatment of 4-HCH significantly improved cardiac systolic dysfunction (decreased EF and FS), suppressed cardiac hypertrophy (increase of heart size, HW/BW, and HW/TL ratios, cross-sectional area of myocytes), and myocardial fibrosis compared with those observed in vehicle-treated mice following Ang II infusion (Fig. 4c–f). These results indicate that the protective effects of 4-HCH against Ang II-induced cardiac remodeling are independent of mechanisms associated with BP reduction.

### 3.4 Network pharmacology analysis identified the target AKT1 and the PI3K-AKT pathways that mediate the effects of 4-HCH against cardiac remodeling

**Fig. 5:**
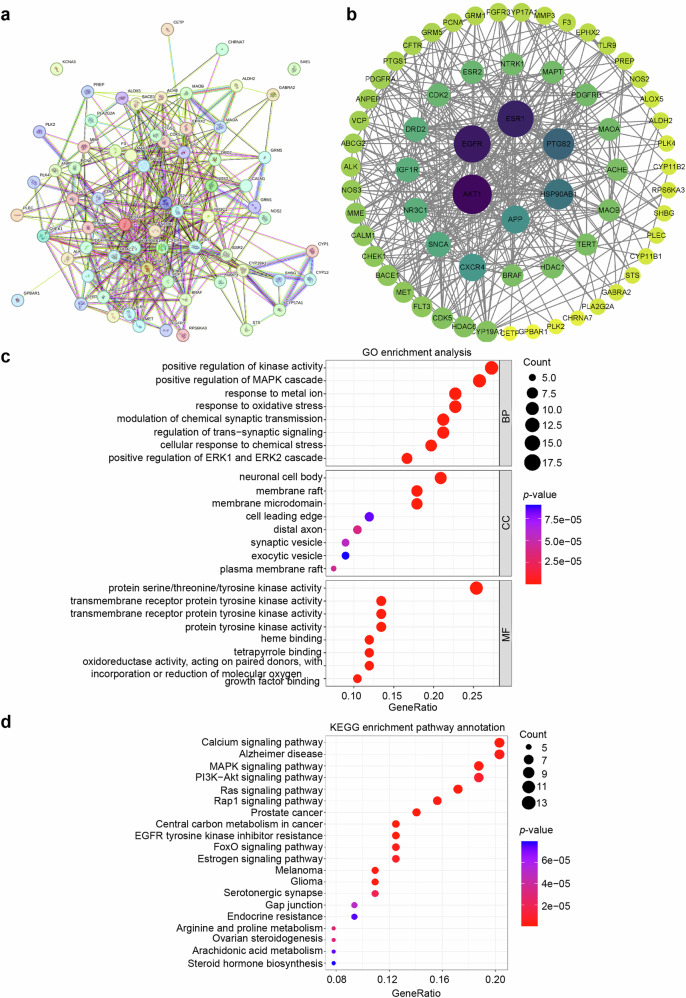
Network pharmacology analysis of 4-HCH and its targets in cardiac remodeling. **a** Network representation of 4-HCH targets in cardiac remodeling. **b** 65 core targets of 4-HCH in cardiac remodeling, with color intensity from yellow to purple indicating degree index. **c** Gene Ontology (GO) enrichment analysis for 4-HCH cardiac remodeling targets, where the X-axis shows gene count and color indicates *P*-value. **d** KEGG enrichment analysis for 4-HCH cardiac remodeling targets, with the X-axis representing gene ratio; bubble size reflects the number of enriched targets, and color indicates *P*-value

A total of 100 targets for 4-HCH were identified using the Swiss Target Prediction database. Concurrently, gene corrections were performed based on UniProt data, and any unmatched components were excluded. Additionally, a total of 16,229 targets related to cardiac remodeling were identified from the GeneCards, OMIM, and DisGeNET databases. Through Venn diagram analysis, 67 overlapping targets between 4-HCH and cardiac remodeling were determined, which may serve as potential therapeutic candidates for treating cardiac remodeling associated with 4-HCH. Subsequently, these overlapping targets of 4-HCH and cardiac remodeling were imported into the STRING tool for protein–protein interaction (PPI) analysis (Fig. 5a, b). Subsequently, these overlapping targets were transferred to Cytoscape for visualization, with the resulting PPI network displayed in Fig. 5b. Notably, the top 10 targets based on degree, betweenness, and closeness centrality consistently included AKT1, EGFR, ESR1, PTGS2, HSP90AB1, APP, CXCR4, SNCA, IGF1R, and DRD2 (Fig. 5b and Supplementary Table 1).

The Hiplot platform facilitated the GO functional enrichment analysis of 67 overlapping targets associated with 4-HCH and cardiac remodeling, yielding a total of 1721 terms. Among these, biological processes (BP), molecular functions (MF), and cellular components (CC) comprised 1429, 128, and 164 items, respectively. The top 8 items from each category with the smallest *P* values were selected for visualization in Fig. 5c. The BP categories indicated that the overlapping targets exhibited enrichment in positive regulation of kinase activity, positive regulation of the MAPK cascade, response to metal ions, and response to oxidative stress (Fig. 5c). In the CC categories, enrichment was observed in neuronal cell body, membrane raft, membrane microdomain, and cell leading edge (Fig. 5c). Furthermore, the overlapping targets in the MF categories were associated with protein serine/threonine/tyrosine kinase activity, transmembrane receptor protein tyrosine kinase activity, transmembrane receptor protein tyrosine kinase activity and protein tyrosine kinase activity (Fig. 5c).

KEGG signaling pathway enrichment analysis was conducted on 67 overlapping targets of 4-HCH and cardiac remodeling using the Hiplot platform. The top 20 identified signaling pathways are displayed in Fig. 5d. Enriched pathways included calcium signaling pathway, alzheimer disease, MAPK signaling pathway and PI3K − AKT signaling pathway (Fig. 5d). These findings indicate that 4-HCH mitigates cardiac remodeling through multiple components and targets.

### Effects of 4-HCH on the expression of PI3K-AKT signaling pathway in the heart after Ang II infusion

**Fig. 6:**
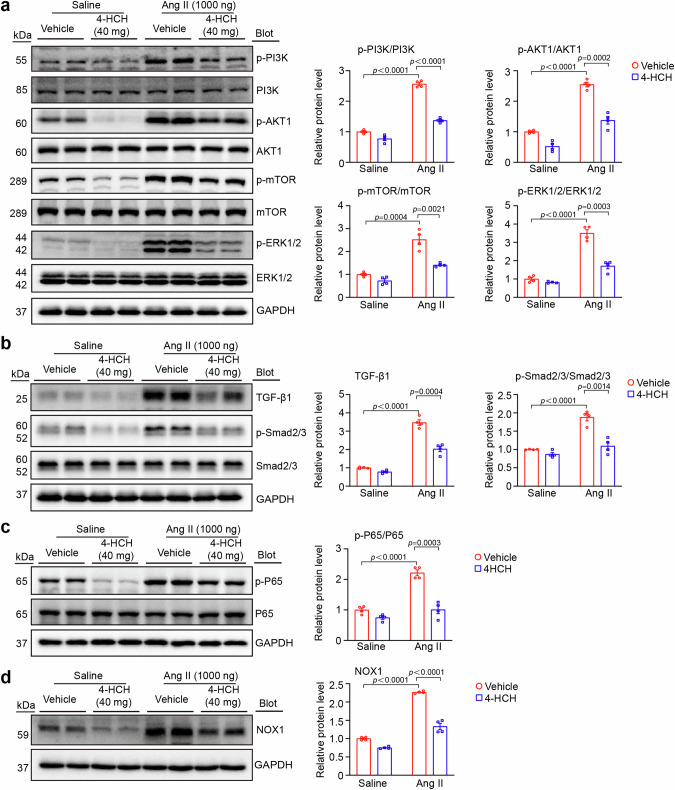
Administration of 4-HCH blunts the activation of multiple signaling pathways. **a** Representative immunoblots of p-PI3K, PI3K, p-AKT1, AKT1, p-mTOR, mTOR, p-ERK1/2, and ERK1/2 in heart tissues from each group (left). Quantification of relative protein levels by densitometry (right, *n* = 4). **b** Representative immunoblots of TGF-β1, p-Smad2/3, and Smad2/3, along with quantification of relative protein levels by densitometry (right, *n* = 4). **c** Representative immunoblot analyses of p-P65 and P65 (left) and quantification of relative protein levels by densitometry (right, *n* = 6). **d** Representative immunoblots of NOX1 (left) and quantification of relative protein levels by densitometry (right, *n* = 4). Data are presented as mean ± SEM, with n representing the number of animals. *P* < *0.05* was considered statistically significant

To determine whether the anti-cardiac remodeling effects of 4-HCH inhibit the activation of the PI3K-AKT signaling pathway, we evaluated the expression levels of PI3K, AKT1, and their downstream proteins. Immunoblotting analysis showed that Ang II-induced upregulation of the protein expression of p-PI3K and p-AKT1, which was markedly decreased in 4-HCH-treated mice (Fig. 6a). We subsequently investigated the effects of 4-HCH on hypertrophic signaling pathways. As expected, the protective effect of 4-HCH against cardiac hypertrophy by reducing the activation of p-mTOR and p-MAPK (ERK1/2) (Fig. 6a).

In addition, 4-HCH treatment significantly inhibited the activation of TGF-β-Smad2/3, NADPH oxidase 1 (NOX1), and NF-κB-p65 pathways in cardiac tissues compared to the vehicle control following Ang II infusion (Fig. 6b–d). No significant differences were observed in the expression of proteins associated with cardiac hypertrophy, fibrosis, inflammation, or oxidative stress signaling pathways in saline-treated controls (Fig. 6a–d).

## Discussion

The present study provides novel evidence indicating that 4-HCH, a class of naturally occurring compounds, can significantly attenuate Ang II-induced hypertension, cardiac remodeling and improve dysfunction following Ang II infusion in mice. Network pharmacology analysis identified the PI3K-AKT pathways as mediators of the effects of 4-HCH against cardiac remodeling. Experimental validation demonstrated that 4-HCH inhibited the activation of the PI3K/AKT1/mTOR/ERK, TGF-β/Smad2/3, NF-κB, and NOX1 signaling pathways, thereby improving cardiac remodeling and dysfunction independently of its BP-lowering effects. To the best of our knowledge, this study is the first to highlight the significant role of 4-HCH in regulating cardiac remodeling and dysfunction.

Ang II infusion induces changes in cardiac cells, molecules, and morphology, leading to maladaptive cardiac responses, progressive remodeling, and dysfunction, ultimately resulting in heart failure (HF) [19]. This response involves a range of mechanisms, including the activation of the sympathetic nervous system, edema, oxidative stress, and inflammation [19, 20]. Despite the availability of pharmacological treatments for cardiac remodeling and HF, including newly recommended angiotensin receptor/neprilysin inhibitors, sodium-glucose cotransporter-2 inhibitors, and vericiguat, the long-term prognosis remains bleak [21]. Therefore, there is an increasing interest in identifying natural products for the prevention and treatment of cardiovascular diseases, as well as in exploring novel therapeutic targets. Natural chalcones are recognized for their diverse biological activities and favorable safety profile. 4-HCH, an α,β-unsaturated ketone with a chalcone core structure, has exhibited a broad spectrum of pharmacological effects, including anti-microbial, anti-inflammatory, proteasome-inhibitory, antioxidant, and anti-angiogenic activities [9–11, 22]. In prior animal studies, the effective therapeutic dose of 4HCH ranged from 10 mg/kg body weight to 40 mg/kg body weight and was deemed to exhibit no significant toxicity [10, 11, 22, 23]. Our findings further indicate that 4-HCH at a dosage of 40 mg/kg body weight demonstrates a favorable safety profile; however, comprehensive toxicity studies, including assessments of both acute and chronic exposure, are warranted in future research. 4-HCH has demonstrated promising clinical applications in medical research, including the treatment of renal injury, allergic asthma, and anti-cancer properties [10, 11, 23]. In this study, we first introduced 4-HCH into the realm of cardiovascular research and demonstrated that this chalcone provides significant benefits in preventing Ang II-induced cardiac remodeling and dysfunction. However, despite these encouraging prospects, the clinical application must undergo a rigorous series of clinical trials to validate its efficacy and safety.

Since the integrated model of network pharmacology indicated that the AKT1 and PI3K-AKT signaling pathway serve as mediators of the effects of 4-HCH in combating cardiac remodeling. Previous studies have demonstrated that the PI3K-AKT signaling pathway regulates the occurrence and progression of cardiac remodeling by influencing myocardial hypertrophy, fibrosis, oxidative stress, and inflammation [24, 25]. Cardiac hypertrophy is an adaptive compensatory mechanism that helps maintain cardiac output in response to harmful stimuli, which can be initiated through two pathways: mTOR and MAPK (ERK), both of which are downstream effectors of AKT [26, 27]. Meanwhile, the TGF-β/Smad pathway, which functions downstream of AKT, plays a crucial role in regulating the development and progression of cardiac fibrosis. This pathway is directly or indirectly linked to the modulation of cardiac fibroblasts and/or the extracellular matrix (ECM) [28]. Oxidative stress and inflammation are two phenomena directly involved in cardiac remodeling, primarily regulated by the NF-κB and NADPH oxidase signaling pathways [29]. In the present study, Ang II was found to induce myocardial hypertrophy, fibrosis, oxidative stress, and inflammation, as evidenced by alterations in the PI3K/AKT1/mTOR/ERK, TGF-β/Smad2/3, NF-κB, and NOX1 signaling pathways (Fig. 6). In contrast, Treatment with 4-HCH abolished the adverse effects of Ang II on cardiac remodeling by inhibiting the activation of the aforementioned signaling pathways (Fig. 6). Additionally, 4-HCH also downregulated PI3K-AKT and its downstream signaling pathway (Fig. 6), but had no significantly effects on cardiac function, hypertrophy, fibrosis, oxidative stress, and inflammation compared to vehicle treatment in saline-treated controls (Figs. 1–3). All these results suggest that the potential mechanism by which 4-HCH alleviates Ang II-induced cardiac remodeling is linked to the activation of the PI3K-AKT pathways, but not under basal conditions.

## Conclusions

In summary, this study represents the first introduction of 4-HCH into cardiovascular research and demonstrates that treatment with 4-HCH can attenuate Ang II-induced cardiac remodeling and dysfunction by modulating the PI3K-AKT pathway independently of its BP-lowering effects. However, further investigation is warranted to explore the clinical applications of 4-HCH.

## Supplementary information

Supplementary Information
