# Supplementary material for: 4-Hydroxychalcone attenuates AngII-induced cardiac remodeling and dysfunction via regulating PI3K/AKT pathway
Source: Hypertens Res. 2024 Dec 24;48(3):1054–67. doi: 10.1038/s41440-024-02068-w (PMC11879844; doi:10.1038/s41440-024-02068-w)
Supplement: Supplementary file 1 — Supplementary Information [file 41440_2024_2068_MOESM1_ESM.docx]

Table 1: PPI analysis of the top 10 common targets with the highest degree values.

| Gene symbol | Degree centrality | Betweenness centrality | Closeness  centrality |
| --- | --- | --- | --- |
| AKT1 | 44 | 0.150720212 | 0.761904762 |
| EGFR | 40 | 0.142135516 | 0.727272727 |
| ESR1 | 39 | 0.135375761 | 0.719101124 |
| PTGS2 | 30 | 0.063537517 | 0.653061224 |
| HSP90AB1 | 28 | 0.031323711 | 0.62745098 |
| APP | 24 | 0.071031993 | 0.60952381 |
| CXCR4 | 23 | 0.02614786 | 0.603773585 |
| SNCA | 19 | 0.032249659 | 0.571428571 |
| IGF1R | 18 | 0.014266107 | 0.561403509 |
| DRD2 | 18 | 0.033952362 | 0.576576577 |
